# Improvement of Fracture Toughness in Epoxy Nanocomposites through Chemical Hybridization of Carbon Nanotubes and Alumina

**DOI:** 10.3390/ma10030301

**Published:** 2017-03-16

**Authors:** Muhammad Razlan Zakaria, Muhammad Helmi Abdul Kudus, Hazizan Md. Akil, Mohd Hafiz Zamri

**Affiliations:** 1 School of Materials and Mineral Resources Engineering, Engineering Campus, University Sains Malaysia, 14300 Nibong Tebal, Pulau Pinang, Malaysia; razlan.usm@gmail.com (M.R.Z.); ibnuquddus@gmail.com (M.H.A.K.); hafiz_zamri@yahoo.com (M.H.Z.); 2 Cluster for Polymer Composite (CPC), Science and Engineering Research Center, Engineering Campus, University Sains Malaysia, 14300 Nibong Tebal, Malaysia

**Keywords:** polymer nanocomposites, carbon nanotubes, hybrid, chemical vapour deposition

## Abstract

The current study investigated the effect of adding a carbon nanotube–alumina (CNT–Al_2_O_3_) hybrid on the fracture toughness of epoxy nanocomposites. The CNT–Al_2_O_3_ hybrid was synthesised by growing CNTs on Al_2_O_3_ particles via the chemical vapour deposition method. The CNTs were strongly attached onto the Al_2_O_3_ particles, which served to transport and disperse the CNTs homogenously, and to prevent agglomeration in the CNTs. The experimental results demonstrated that the CNT–Al_2_O_3_ hybrid-filled epoxy nanocomposites showed improvement in terms of the fracture toughness, as indicated by an increase of up to 26% in the critical stress intensity factor, *K*_1*C*_, compared to neat epoxy.

## 1. Introduction

The world of composite materials is expanding due to its new potential of building various kinds of hybrid compounds. These hybrid compounds provide intriguing challenges and opportunities for chemists, physicists, biologists, engineers, and material scientists [1,2]. The hybrid concept is a simple extension of the composite principle of combining more than one reinforcement material. The idea behind having a hybrid compound is to combine the properties of a few materials into a single material to enable engineers to utilize the full potential of the synergistic effect of the combination. The use of hybrid compounds not only helps to enhance the functionality of conventional materials, but also offers remarkable possibilities, together with lower costs and more environmentally-friendly production methods, for the newly-developed materials in scientific and technological applications [3]. Many hybrid compounds have contributed to the scientific and technological applications of polymer composites. Among the hybrid compounds, carbon nanotube (CNT)–inorganic hybrids are a new and promising class of functional materials that offer a synchronized combination of a nano–micrometre sized reinforcement and the functionality of an organic–inorganic framework [4]. For instance, studies on CNT–ceramics have shown that the hybrid compound gives exceptional performance in several applications, such as superior activities in photocatalysts (CNT–TiO_2_) [5,6], enhanced capacities in super capacitors (CNT–MnO_2_) [7], improved efficiency in photovoltaic cells (CNT–ZnO) [8], and increased sensitivity in gas sensors (CNT–SnO_2_) [9].

Recently, CNTs have been hybridized with alumina (Al_2_O_3_) particles and used as filler in polymer composites [10,11,12]. The properties of Al_2_O_3_—such as its high level of hardness, refractoriness, excellent dielectric and good thermal properties—make it a suitable choice for hybridization with CNTs [13]. A few hybridization methods have been reported previously, such as milling and hot press sintering. However, both of these methods cause damage to the CNT structure due to the long milling duration and the influence of a high compression force. In addition, these methods also cause non-uniform dispersion of the CNTs and Al_2_O_3_ particles, leading to agglomeration of the CNTs during the fabrication of the polymer composites. To overcome this problem, the CNTs and Al_2_O_3_ were hybridized via chemical vapour deposition (CVD) to improve the dispersion of the CNTs without damaging their structure and to maintain the properties of the CNTs. Furthermore, other advantages of CVD are its simplicity and ability to produce massive quantities of CNTs [14].

Epoxy resins are categorized as an interesting class of polymers with a wide range of applications in coating, moulding, adhesives, and composites. Epoxy resins can be cured with various types of curing agents and are usually brittle and stiff due to their high cross-link density, thereby leading to low toughness. There are several techniques for overcoming these disadvantages, such as blending the epoxy with ductile and flexible polymers or adding fillers such as thermoplastics, rubber, or rigid particles. Usually, the modulus and strength are decreased when the toughness is increased. However, in some situations, the toughness is improved without any loss in strength and modulus when nano particles are used as the filler.

In this paper, a CNT–Al_2_O_3_ hybrid compound was used as filler to improve the toughness of epoxy nanocomposites. The CNT–Al_2_O_3_ hybrid compound was synthesized by growing the CNTs on the Al_2_O_3_ particles via CVD. The characteristics of the CNT–Al_2_O_3_ hybrid compound and the physically-mixed CNT–Al_2_O_3_ were examined using a field emission scanning electron microscope (FESEM) and a high-resolution transmission electron microscope (HRTEM) to observe the morphologies of the CNTs and Al_2_O_3_ particles. The aim of this study was to investigate the fracture toughness of epoxy nanocomposites. The dispersion of the CNTs in the epoxy nanocomposites were examined using FESEM and HRTEM. Possible explanations for the difference between the CNT–Al_2_O_3_ hybrid compound-filled epoxy and the physically-mixed CNT–Al_2_O_3_-filled epoxy will be discussed.

## 2. Experimental Procedures

A CNT–Al_2_O_3_ hybrid compound was synthesized using the chemical vapour deposition (CVD). The catalyst was prepared by precipitating nickel nitrate hexahydrate (Ni(NO_3_)_2_·6H_2_O) on aluminium (Al) powder in a solution of sodium hydroxide (NaOH). The catalyst was then dried at 110 °C for 2 h and calcined at 900 °C to form a NiO–Al_2_O_3_ complex. The NiO–Al_2_O_3_ complex was subjected to a reduction process under hydrogen gas at 400 °C for 2 h, followed by the growth of the CNTs onto the Al_2_O_3_ particles under a methane and nitrogen gas atmosphere at a ratio of 1:7 at 800 °C for 30 min in a horizontal tube furnace. The reagent grade Ni(NO_3_)_2_·6H_2_O, Al powder, and NaOH were obtained from Merck & Company. CNTs and Al_2_O_3_ were physically mixed using a ball milling machine for 48 h at 20 rpm for comparison with the CNT–Al_2_O_3_ hybrid compound. Pure CNT (supplied by Sky Spring Nanomaterials Inc., Houston, TX, USA, with 95% purity) and Al_2_O_3_ (supplied by Sigma Aldrich, Saint Louis, MO, USA, with 98% purity) were mixed at a ratio of 12:100 based on the energy dispersive X-ray (EDX, ZEISS, Oberkochen, Germany) analysis reported in a previous paper [15].

The morphologies of the CNT–Al_2_O_3_ hybrid compound and the physically-mixed CNT–Al_2_O_3_ were analysed using a field emission scanning electron microscope (SEM, Leo Supra-35VP, ZEISS) and a high-resolution transmission electron microscope (HRTEM, Philip TECNAI 20, FEI, Hillsboro, OR, USA).

1.0, 3.0, and 5.0 wt % of the CNT–Al_2_O_3_ hybrid compound and physically mixed CNT–Al_2_O_3_ were dispersed in epoxy resin diglycidyl ether of bisphenol A (DGEBA) using a sonicator (Q700, Qsonica, Melville, CT, USA) at a frequency of 25 kHz for 30 min. The curing agent trimethylhexamethylenediamine (TMD) was then added to the mixture with a mass ratio to epoxy resin of 6:10. The mixture was placed in a vacuum at 76 cm Hg pressure for 30 min to remove any trapped air, and was then poured into a silicon mould. Finally, the epoxy composites were cured at 120 °C for 1 h. Table 1 shows the description of the samples.

**Figure 1:**
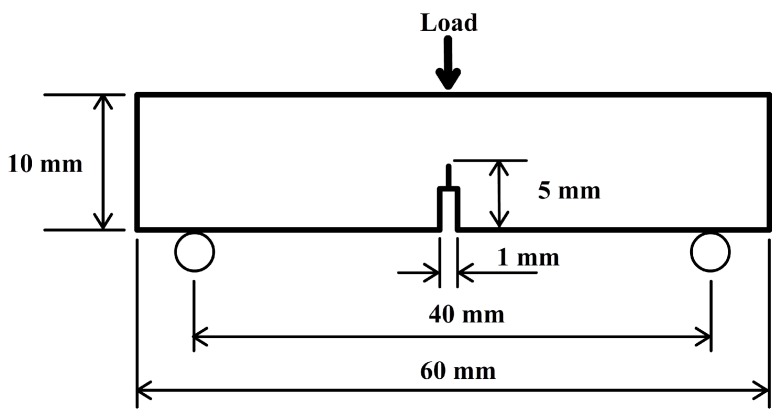
Single-edge notch three-point bending (SEN-3PB) specimen geometry used for the fracture toughness.

Single-edge notch three-point bending (SEN-3PB) tests were conducted to obtain the fracture toughness in terms of the critical stress intensity factor, *K*_1*C*_, for the epoxy nanocomposites according to the ASTM D5045 standard [16]. The tests were conducted with a universal testing machine model (Instron machine type 5960). The dimensions of each sample were 5 mm × 10 mm × 60 mm with a 5-mm notch in the centre of the sample, as shown in Figure 1. The tests were conducted with a load cell of 100 kN and a span length of 40 mm at a crosshead speed of 1 mm/min. In order to avoid the influence of the processing procedure, at least five specimens were tested to ensure the reliability of the test results. According to the standard used, *K*_1*C*_ was calculated using Equation (1):

(1) $$K_{1C}=\frac{6P}{BW}\times a^{\frac{1}{2}}\times Y$$

where Y is the shape factor determined by using Equation (2):

(2) $$Y=\frac{1.99-\frac{a}{W}\left(1-\frac{a}{W}\right)\left(2.15-3.93\frac{a}{W}+2.7{\left(\frac{a}{W}\right)}^{2}\right)}{\left(1+2\frac{a}{W}\right){\left(1-\frac{a}{W}\right)}^{\frac{3}{2}}}$$

where *P* is the maximum load; *B* is the specimen thickness; *W* is the specimen width; and a is the total notch length.

The fracture surface of the epoxy nanocomposites was analysed using FESEM after coating with a 5–10 nm layer of Au-Pd by sputtering. The nano-scale morphologies of the Epoxy/HYB and Epoxy/MIX were analysed using HRTEM. The HRTEM samples with a thickness of 50 nm were prepared by cryo-ultramicrotomy using a microtome by Leica (Reichert-Jung Ultracut E, Leica Microsystems, Wetzlar, Germany).

## 3. Results and Discussion

**Figure 2:**
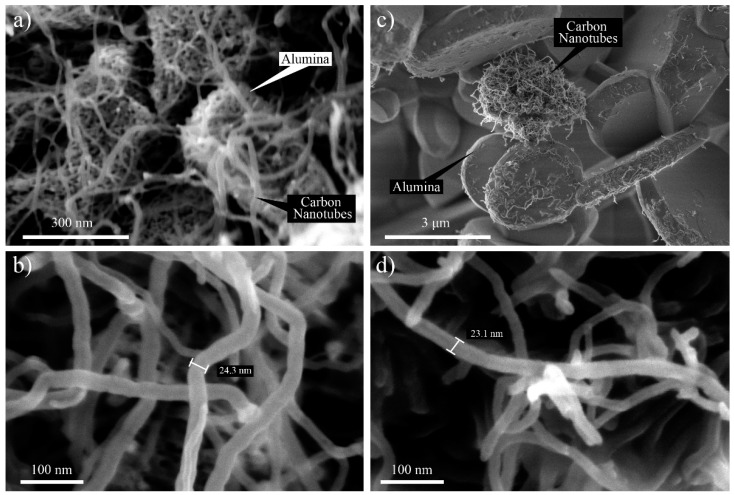
Field emission scanning electron microscopy (FESEM) images of HYB with magnification of (**a**) 200,000× and (**b**) 350,000× and MIX with magnification of (**c**) 40,000× and (**d**) 350,000×.

FESEM was used at different magnifications ranging from 40,000× to 35,000× to investigate the morphological aspects of the HYB and MIX. The SEM images of the HYB are shown in Figure 2a,b. It could be clearly observed that the CNTs were successfully deposited onto the Al_2_O_3_ particles. In addition, the CNTs were attached and distributed around the Al_2_O_3_ particles. The diameter of the grown CNT was approximately 10–30 nm. Meanwhile, the SEM image of the MIX illustrated poor distribution of the CNTs and Al_2_O_3_ particles (as shown in Figure 2c,d). The CNTs and Al_2_O_3_ particles did not appear to be attached together compared to the HYB. The CNTs tended to form bundles due to the van der Waals interactions [17].

**Figure 3:**
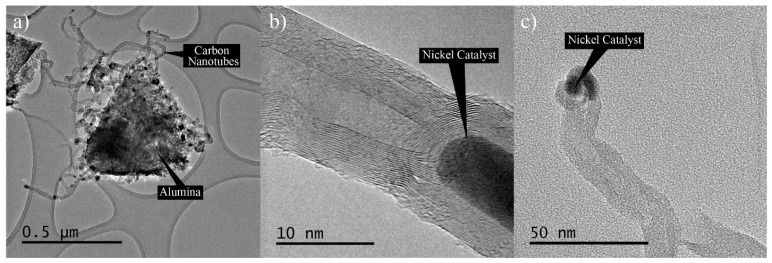
High-resolution transmission electron microscopy (HRTEM) images of HYB.

The morphology of the HYB was analysed using HRTEM in order to have a close-up view of the nano-scale morphology. Figure 3a shows that the HYB was in the form of wire-like hollow structures, as seen under SEM. Figure 3b shows the hollow structure and the multi-layered wall, which was confirmed to be multi-walled carbon nanotubes (MWCNTs). The MWCNTs contained several layers of graphene sheets along the longitudinal direction of the nanotubes with approximately 15–30 walls. The nickel catalyst could be clearly observed at the tips of the MWCNTs (as shown in Figure 3c), which indicated that the MWCNTs had grown by the tip growth mode. This observation seemed to be in agreement with several reports [18,19], where the small particles on the tips of the CNTs were metal catalyst particles. During the decomposition of methane in CVD, the nickel particles behaved as a metal catalyst to grow the CNTs onto the Al_2_O_3_ particles in appropriate conditions. At an elevated temperature, the nickel particles that had been attached to the surfaces of the Al_2_O_3_ particles melted, and methane started to decompose the carbon. The hotter phase of the molten nickel absorbed the decomposed carbon and rearranged the carbon elements at the colder side of the nickel, and in time formed CNTs. The nickel particles catalysed the growth of the CNTs until the preferred reaction time was reached.

**Figure 4:**
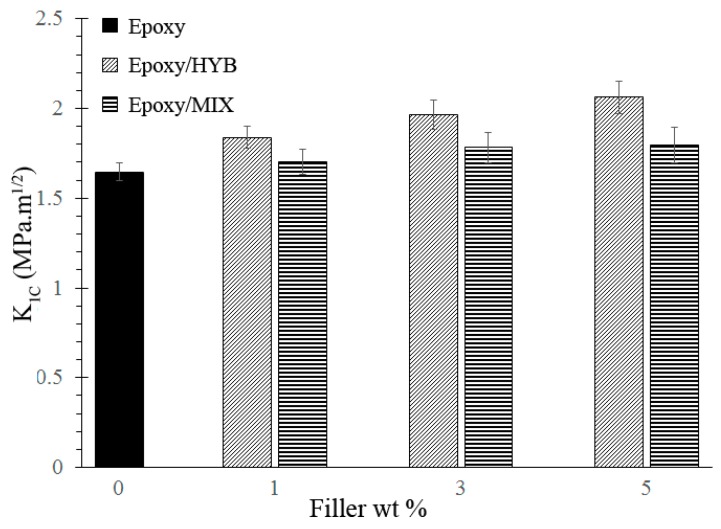
Stress intensity factor (*K*_1*C*_) of the neat epoxy and the epoxy composites with 1 wt %, 3 wt %, and 5 wt % of HYB and MIX.

The fracture toughness of neat epoxy, Epoxy/HYB, and Epoxy/MIX was determined by the stress intensity factor, *K*_1*C*_, which was calculated based on a SEN-3PB experiment using Equation (1), and the results are presented in Figure 4. From the results, it could be seen that there was an improvement in the *K*_1*C*_ following the addition of the HYB, but it was not significant for MIX. The fracture toughness of the Epoxy/HYB approached a maximum at 5 wt % HYB with 2.06 MPa·m^1/2^, representing an increase of over 26% in comparison to that of the neat epoxy. Meanwhile, the fracture toughness of the Epoxy/MIX approached a maximum at 5 wt % MIX with 1.79 MPa·m^1/2^, representing only a slight increase which was not significant compared to that of the neat epoxy. A few factors contributed to the enhancement of the fracture toughness, such as the hybrid filler architecture and morphology of the epoxy nanocomposites. The hybrid filler architecture played a crucial role in improving the fracture toughness of the epoxy nanocomposites. In this epoxy nanocomposite system, the hybrid filler combination consisted of rigid Al_2_O_3_ particles and CNTs which possessed extraordinary strength and modulus, leading to an improvement in the fracture toughness. As reported previously in many studies, CNTs possess a strength and modulus of about 63 GPa and 1000 GPa, respectively, which are far higher than the strength and modulus of neat epoxy [20,21]. Hence, these extraordinary properties of CNTs prevent the epoxy nanocomposites from being easily fractured and enable them to withstand a higher load. Based on Figure 4, it was demonstrated that the Epoxy/HYB composite showed a higher fracture toughness compared to the Epoxy/MIX composite. This was due to the architecture of the HYB, which affected the morphology of the epoxy nanocomposites. In an epoxy composite system, it is important to consider the dispersion state of the filler. The good HYB architecture produced a homogenous dispersion of CNTs in the epoxy matrix. The homogenous dispersion could have been achieved by the Epoxy/HYB, because the Al_2_O_3_ particles helped the CNTs to form a network by preventing the CNTs from agglomerating and simultaneously working to transport the dispersed CNTs. Thus, the CNT network formed by the HYB provided a more effective load transfer between the epoxy matrix and the filler, thereby improving the fracture toughness of the epoxy nanocomposites. In addition, the homogenous dispersion of CNTs also contributed to a wider surface for bonding with the epoxy matrix. A similar trend of the increasing fracture toughness by using CNTs as the filler was also reported by Shokrieh et al. [22]. As discussed in that paper, the CNTs increased the fracture toughness of the composites without reducing other mechanical properties such as the strength and modulus. It could be seen that the fracture toughness of the Epoxy/MIX was slightly lower than that of the Epoxy/HYB because a different hybridization technique was used to produce the MIX. The architecture of the MIX—which was totally different from that of the HYB—affected the final morphology of the epoxy nanocomposites. In the Epoxy/MIX, the architecture of the MIX—which distributed the CNTs and Al_2_O_3_ particles separately—inclined the CNTs to agglomerate due to the van der Waals forces and the high surface area. This agglomeration of CNTs reduced the surface area of the CNTs and introduced stress concentrations, which consequently reduced the ability of the epoxy nanocomposites to withstand the load.

**Figure 5:**
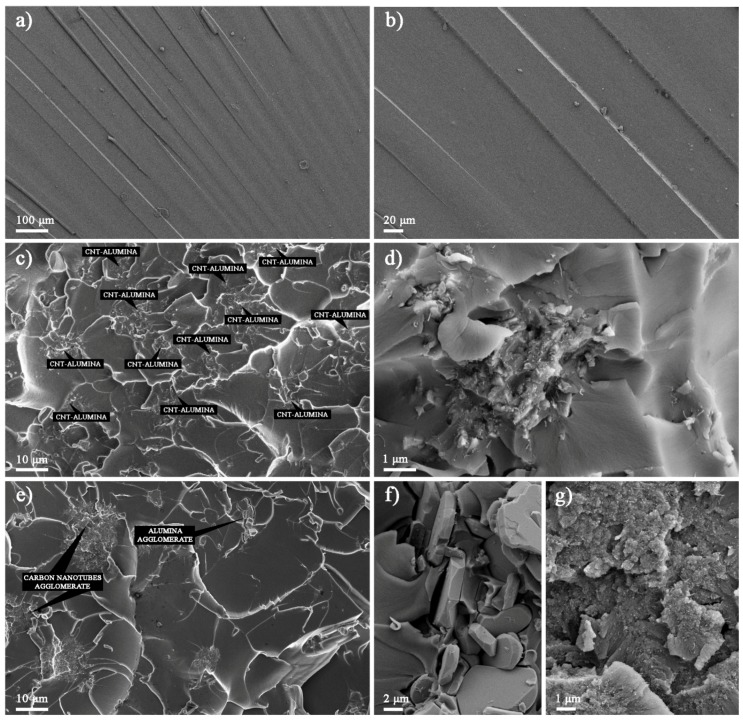
FESEM images of the neat epoxy at magnification of (**a**) 100×; (**b**) 300×; Epoxy/HYB at magnification of (**c**) 1000×; (**d**) 10,000×, and Epoxy/MIX at magnification of (**e**) 1000×; (**f**) 3000×; and (**g**) 5000×.

The fracture surfaces of the Epoxy/HYB and Epoxy/MIX were observed using FESEM in order to understand the improvement in the fracture toughness. It was observed that the fracture surface of the neat epoxy appeared to be smooth, as shown in Figure 5a,b. This fracture surface morphology revealed the brittle nature of the neat epoxy, which had poor facture toughness due to its weak resistance to crack initiation and propagation under load. The fracture surface of the Epoxy/HYB is shown in Figure 5c,d, which illustrates the HYB as being homogenously dispersed in the epoxy matrix. This homogenous dispersion provided a large contact area between the CNTs and the epoxy matrix due to the HYB architecture, which prevented the CNTs from agglomerating. Therefore, more bridging networks were formed, which enabled the load to be transferred from the matrix to the filler. In addition, small bright dots could be seen at the fracture surface, which indicated the ends of the broken CNTs. These CNTs were broken instead of being pulled out of the epoxy matrix, which means that the epoxy matrix was able to tightly hold the CNTs throughout the interfacial bonding, thereby illustrating strong interfacial bonding. Therefore, this confirmed that the hybridization of CNTs can improve the quality of the interfacial bonding and enhance the fracture toughness, as stated earlier. Furthermore, it could be seen that the Al_2_O_3_ particles were evenly surrounded by the CNTs, and the number of unattached CNTs in the epoxy matrix was minimal. The fracture surface of the Epoxy/MIX demonstrated that the MIX was not homogenously dispersed in the epoxy matrix, as shown in Figure 5e–g. From the fracture surface, it could be seen that the CNTs and Al_2_O_3_ particles were separated and some CNTs were detached from the Al_2_O_3_ particles. This was due to the weak interaction bonding between the CNTs and Al_2_O_3_ particles in the MIX as a result of the sonication process at high frequency vibration. The agglomeration of the Al_2_O_3_ particles and the CNTs in the epoxy matrix could be seen clearly from the close-up observation in Figure 5f,g. This agglomeration induced stress concentration, thus reducing the fracture toughness properties.

Based on the surface roughness, it could be seen that the neat epoxy had river stripe marks with minimal deformation. The addition of the HYB and MIX into the epoxy matrix resulted in improved energy dissipation and toughening through the formation of many micro-cracks with cleavage planes on their fracture surfaces. The network of cleavage steps formed the cleavage planes, and each plane consisted of at least CNTs or Al_2_O_3_ particles. The formation of the cleavage planes was due to the change in the direction of the crack propagation as it crossed the CNTs or Al_2_O_3_ particles. Thus, this bridge effect—which prevented the cracks from opening—improved the fracture toughness of the epoxy nanocomposites. The decreased size of the cleavage plane made it more difficult for the cracks to propagate, as the CNTs and Al_2_O_3_ particles distorted the path of the crack tip. It could be seen that the size of the cleavage plane of the Epoxy/HYB was much smaller than that of the Epoxy/MIX. This means that more energy was absorbed by the generation of numerous micro cracks dissipated in the Epoxy/HYB during the fracture process.

**Figure 6:**
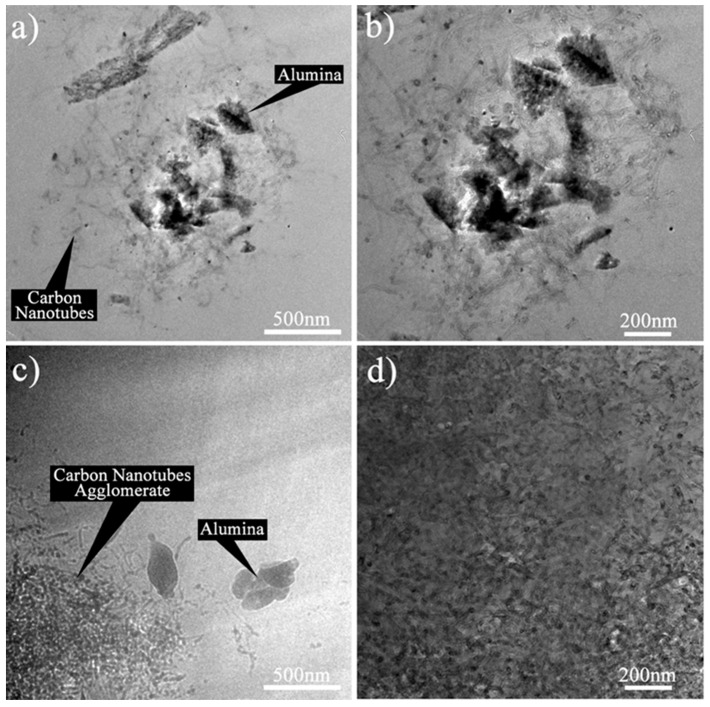
HRTEM images of the Epoxy/HYB at magnification of (**a**) 19,500×; (**b**) 29,000×; and Epoxy/MIX at magnification of (**c**) 19,500×; and (**d**) 29,000×.

HRTEM was carried out to further evaluate the dispersion of the HYB and MIX in the epoxy nanocomposites. The morphologies of the Epoxy/HYB and Epoxy/MIX were examined at magnifications of 19,500× and 29,000×. Figure 6a,b illustrates the HRTEM images of the Epoxy/HYB, and show that the CNTs and Al_2_O_3_ particles were dispersed together in the epoxy matrix. The CNTs were well-dispersed around the Al_2_O_3_ particles, and there was less agglomeration of CNTs. The Al_2_O_3_ particles functioned as transportation for the CNTs to disperse homogenously and to prevent the CNTs from agglomerating. Meanwhile, the Epoxy/MIX demonstrated poor dispersion and agglomeration of the CNTs and Al_2_O_3_ particles, as shown in Figure 6c,d. This was because the Al_2_O_3_ particles appeared to disperse alone instead of helping the CNTs to disperse in the epoxy matrix. Therefore, it could be concluded that the dispersion of the CNTs in the Epoxy/HYB composite was dependent on the Al_2_O_3_ particles, while the dispersion of the CNTs in the Epoxy/MIX was not dependent on the Al_2_O_3_ particles.

**Figure 7:**
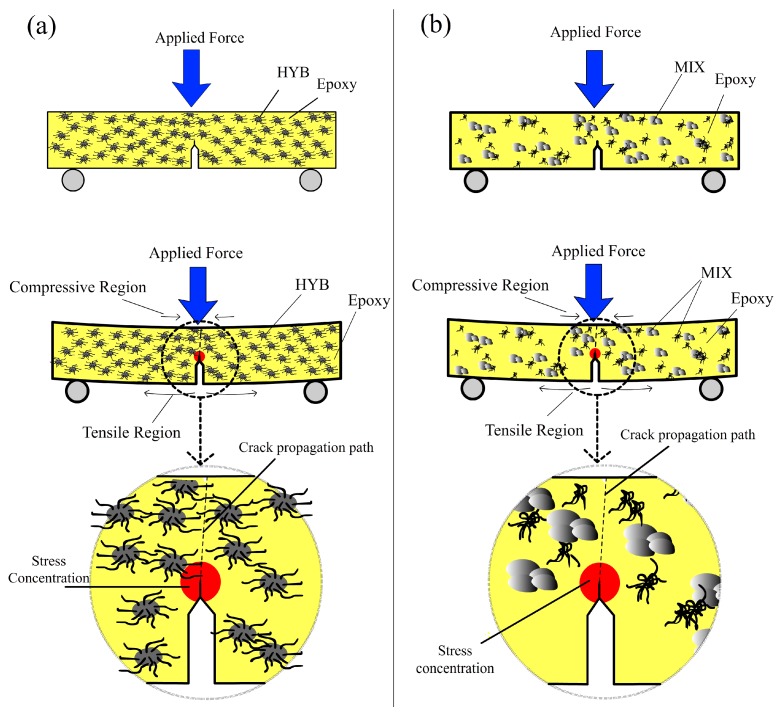
Schematic representation of the crack propagation in the (**a**) Epoxy/HYB and (**b**) Epoxy/MIX.

The failure modes of the epoxy composites were the result of various factors and mechanisms, such as stress transfer between the matrix and the reinforcement, interface debonding, stress concentration points, and crack propagation [23]. The stress conditions in the Epoxy/HYB and Epoxy/MIX, and possible failure modes during the fracture toughness are shown schematically in Figure 7a,b, respectively. It is believed that the epoxy nanocomposites on the compressive region underneath the applied load underwent local crushing, while the epoxy nanocomposites on the tensile region tended to debond and fracture [23]. The notch regions in the samples acted as local stress concentration sites and caused the matrix to crack, eventually leading to failure. The crack was initiated at the tensile region and propagated to the compressive region. The failure of the epoxy nanocomposites under fracture toughness was somehow affected by the presence of the HYB filler but not significantly affected by MIX filler. Since the HYB and MIX fillers used in this study possessed extremely high mechanical strength, the HYB and MIX fillers continued carrying the load when the stress level exceeded the *K*_1*C*_ of the neat epoxy (0.9 MPa·m^1/2^), which resulted in fracturing of the epoxy matrix. The propagation of cracks in the Epoxy/HYB was delayed by the better dispersion of fillers compared to the Epoxy/MIX. As shown in Figure 7b, the crack propagation in the Epoxy/MIX occurred around the agglomerated particles or was due to the weak structure of the epoxy matrix.

## 4. Conclusions

This study investigated the effect of HYB and MIX on the fracture toughness of the epoxy nanocomposites at various filler loadings. Based on the experimental findings, it can be concluded that the incorporation of HYB and MIX in the epoxy composites resulted in an improvement in the fracture toughness of the epoxy nanocomposites. The Epoxy/HYB showed a higher fracture toughness compared to the Epoxy/MIX. The fracture toughness of the Epoxy/HYB was enhanced by about 26%, whereas the fracture toughness of the Epoxy/MIX was enhanced by about 9%. When the fracture surface was observed under FESEM and HRTEM, it could be seen that the HYB was homogenously dispersed and there was some agglomeration of MIX in the epoxy matrix. It can be concluded that the better dispersion of HYB in the epoxy matrix led to higher fracture toughness.

## Figures and Tables

**Table 1:** Shows the description of the samples.

Samples	Decriptions
HYB	CNT–Al_2_O_3_ hybrid compound
MIX	CNT–Al_2_O_3_ physically mixed
Epoxy/HYB	CNT–Al_2_O_3_ hybrid compound filled epoxy nanocomposites
Epoxy/HYB1	1 wt % CNT–Al_2_O_3_ hybrid compound filled epoxy nanocomposites
Epoxy/HYB3	3 wt % CNT–Al_2_O_3_ hybrid compound filled epoxy nanocomposites
Epoxy/HYB5	5 wt % CNT–Al_2_O_3_ hybrid compound filled epoxy nanocomposites
Epoxy/MIX	CNT–Al_2_O_3_ physically mix filled epoxy nanocomposites
Epoxy/MIX1	1 wt % CNT–Al_2_O_3_ physically mix filled epoxy nanocomposites
Epoxy/MIX3	3 wt % CNT–Al_2_O_3_ physically mix filled epoxy nanocomposites
Epoxy/MIX5	5 wt % CNT–Al_2_O_3_ physically mix filled epoxy nanocomposites
